# Policy: Live from Dubai: A New Chemical Agreement

**Published:** 2006-06

**Authors:** Valerie J. Brown

After late-night, last-minute negotiations, a voluntary international agreement to protect humans and the environment against harmful chemicals was adopted on 6 February 2006. Representatives from 140 countries, environmental advocacy groups, industry associations, and UN agencies attended the three-day International Conference on Chemicals Management (ICCM) in Dubai, United Arab Emirates. The agreement establishes the Strategic Approach to International Chemicals Management (SAICM), which gives nations a framework for fulfilling the 2002 World Summit on Sustainable Development goal of ensuring that chemicals are produced and used in ways that minimize significant adverse effects.

Implementation of SAICM will be supported by a new chemicals secretariat within the UN Environment Programme that will carry out the “Overarching Policy Strategy.” This strategy provides countries, especially economies in transition, with templates to begin coping with issues such as remediating contamination, using safer substitutes, and creating toxic release inventories. The agreement offers broad suggestions such as reducing exposures by improving occupational safety, developing better responses to spills and accidents, and eliminating child labor involving chemicals. Some EU countries offered modest funding for a “Quick Start Programme” to help developing countries move ahead in the near term.

Many participants saw the meeting as polarized into EU and U.S. camps on some of the most contentious issues, including precaution—regulating or banning chemicals suspected of harm without complete certainty of their effects. In a February 7 statement on behalf of the EU presidency, Austrian minister Josef Pröll said, “We don’t need to see a tragedy happen to put safety systems in place.” The same day, U.S. assistant secretary of state Claudia McMurray told an AP reporter, “We have a different approach to the way we regulate chemicals in our country. We may not know everything now, but let’s move forward anyhow.”

The agreement incorporates wording from the 1992 Rio Declaration on Environment and Development stating that precaution “shall be widely applied by States according to their capabilities. Where there are threats of serious or irreversible damage, lack of full scientific certainty shall not be used as a reason for postponing cost-effective measures to prevent environmental degradation.” The EU pushed to elaborate on this statement with a clearer connection between chemicals and human health—a push the U.S. delegation opposed.

Disagreement also arose over whether the agreement should invoke specific international bodies such as the World Bank or the Global Environment Facility as potential funding sources. The United States failed to win language that would have made SAICM irrelevant to multinational regulations such as those of the World Trade Organization, a position some say was aimed at keeping environmental and human health values from challenging trade practices.

In a February 27 press release McMurray said, “SAICM recognizes that while we all share the goal of minimizing the risks presented by some chemicals, there are many valid ways to achieve that goal.” But others saw the meeting as lacking political will. Daryl Ditz, senior policy adviser for chemicals at the Washington, DC–based NGO Center for International Environmental Law, says, “Regrettably, the United States was the number-one obstacle to a coordinated global response to the problems posed by chemicals.”

The ICCM next revisits the Dubai agreement in 2009 to assess progress and identify problems.

